# Research of Maritime Object Detection Method in Foggy Environment Based on Improved Model SRC-YOLO

**DOI:** 10.3390/s22207786

**Published:** 2022-10-13

**Authors:** Yihong Zhang, Hang Ge, Qin Lin, Ming Zhang, Qiantao Sun

**Affiliations:** College of Information Science and Technology, Donghua University, Shanghai 201620, China

**Keywords:** YOLOv4-tiny, object detection, receptive field block, convolutional block attention module

## Abstract

An improved maritime object detection algorithm, SRC-YOLO, based on the YOLOv4-tiny, is proposed in the foggy environment to address the issues of false detection, missed detection, and low detection accuracy in complicated situations. To confirm the model’s validity, an ocean dataset containing various concentrations of haze, target angles, and sizes was produced for the research. Firstly, the Single Scale Retinex (SSR) algorithm was applied to preprocess the dataset to reduce the interference of the complex scenes on the ocean. Secondly, in order to increase the model’s receptive field, we employed a modified Receptive Field Block (RFB) module in place of the standard convolution in the Neck part of the model. Finally, the Convolutional Block Attention Module (CBAM), which integrates channel and spatial information, was introduced to raise detection performance by expanding the network model’s attention to the context information in the feature map and the object location points. The experimental results demonstrate that the improved SRC-YOLO model effectively detects marine targets in foggy scenes by increasing the mean Average Precision (mAP) of detection results from 79.56% to 86.15%.

## 1. Introduction

In recent years, as countries around the world attach importance to marine resources and development, a series of emerging marine industries have continued to rise. At the same time, due to the rapid development of the marine economy and the complex and diverse marine environments, the frequency of maritime accidents also increases. As an important branch of object detection, marine object detection is of great significance for maritime navigation, marine environment detection, and even national defense security [1]. However, at present, a complete rescue system has not yet been formed to achieve efficient maritime search and rescue, and a lot of manpower and material resources are still expended in responding to emergencies. Additionally, the complex marine conditions, such as storm surges, waves, fog, and other factors, greatly increase the challenge of rescue. The emergency search and rescue system still requires further improvement [2].

How to overcome the complexity and uncertainty of the marine conditions, detect the target location quickly, and accurately and achieve timely tracking has become a research focus for domestic and foreign scholars. For example, Lang et al. [3] proposed a new scheme for detecting ship targets in high-resolution single-channel synthetic aperture radar (SAR) images, which significantly enhanced the separability between ship targets and sea clutter. In addition, Singh et al. [4] devised a method to identify the ship’s position in the ocean by estimating global thresholding, but only limited to the static position of the ship. With the continuous development of deep learning, intelligent search and rescue based on computer vision has gradually emerged as a trend for marine rescue. Mou et al. [1] introduced a marine target detection method based on improved Faster R-CNN for navigation radar PPI images, but its detection speed still needs to be improved. Meanwhile, a CFAR algorithm was employed to improve the performance of Faster R-CNN in multiscale ship detection tasks in Kang et al. [5]. However, the detection of small targets cannot be achieved well.

In addition, quantifying uncertainty in high-security domains is critical because relying exclusively on deep models for decision-making has the potential to lead to catastrophic consequences. Kendall [6] presented a Bayesian deep learning framework combining input-dependent aleatoric uncertainty together with epistemic uncertainty. Additionally, De Sousa Ribeiro et al. [7] proposed both a Deep Bayesian Self-Training methodology for automatic data annotation, highlighting the importance of predictive uncertainty estimates in safety-critical domains.

Because of the impact of complicated surroundings, such as foggy surfaces and sunlight reflection, the original images can be enhanced through preprocessing. In the meantime, the rapid development of object detection also provides guarantees for efficient sea rescue. At the present stage, the detection methods can be divided into the following two types. The first type is the two-stage model represented by algorithms such as SPP-Net [8], R-CNN [9], and Faster-RCNN [10], which first generates region proposals where targets are likely to appear and then classifies and regresses each region proposal separately. The other category is the one-stage approach represented mainly by the EfficientDet [11], RetinalNet [12], and YOLO [13] series, which transforms the task of object detection into a regression problem for analysis and outputs the detection results directly in an end-to-end manner [14,15,16,17]. At present, object detection based on deep learning has been more widely applied in the fields of face recognition, industrial inspection, and intelligent transportation. In this paper, an improved model SRC-YOLO is proposed based on YOLOv4-tiny, which greatly improves the accuracy of maritime target detection in foggy environments.

As a lightweight model of YOLOv4 [18], YOLOv4-tiny is a state-of-the-art object detection algorithm with the advantages of fewer parameters and faster speed to ensure realtime accuracy of detection tasks. In particular, it makes it possible for UAV clusters to integrate a deep learning framework for efficient ocean search and rescue. The network structure of YOLOv4-tiny is mainly composed of the following three parts. Firstly, the backbone network replaces the full connection by convolution operation for feature extraction. In addition, the Feature Pyramid Networks (FPN) [19] is adopted to achieve multiscale feature fusion. Finally, the detection result of the target is output through the detection module.

Our proposed SRC-YOLO model retains the advantages of fewer parameters and high accuracy, which greatly improves the performance of maritime target detection in foggy environments. The improved RFB_sim and CBAM introduced in the model effectively enhance the performance of the receptive field and detection of small targets. However, there are still some problems that need to be further improved in future work. For example, it is necessary to propose an improved algorithm to remove not only the effect of fog in the image but also the effect of other noises, such as rain and snow.

The main contributions of this work are summarized as follows:

- In the Neck part of the YOLOv4-tiny model, we applied an improved RFB_sim model instead of the standard convolution, which not only enhances the receptive field of the model but also improves the performance of small object detection on the basis of data enhancement using the SSR algorithm.
- Through the comparative analysis of different attention mechanisms, we introduced the CBAM that combines the channel and spatial information to improve the focus on the targets in the output part of feature extraction and visualized the feature extraction results by Class Activation Mapping (CAM).

## 2. Related Work

### 2.1. SRC-YOLO Model Structure

**Figure 1:**
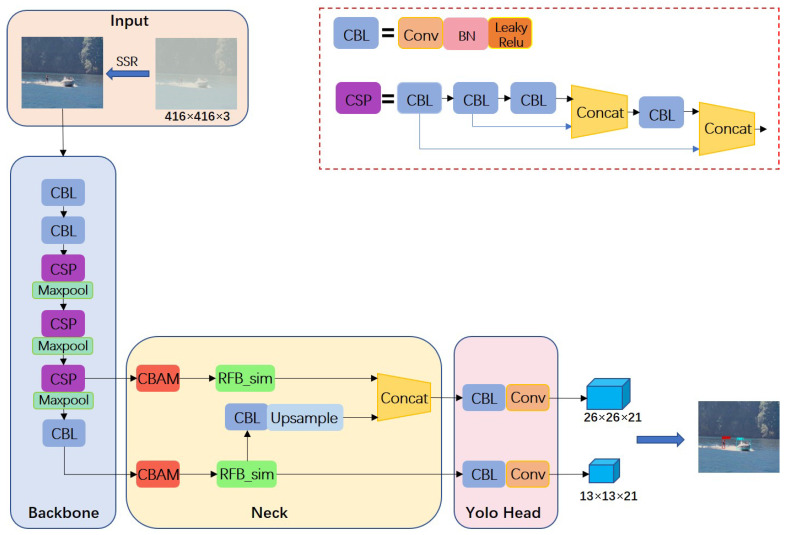
The network structure SRC-YOLO.

In order to preserve the efficient feature extraction performance of YOLOv4-tiny, the Backbone network, composed of CBL and CSPBlock modules with each other, still adopts the CSPMarket53-tiny structure in the YOLOv4-tiny model. In particular, the CBL utilizes LeakyReLU as the activation function after convolution to improve the detection speed of the model. Meanwhile, there are two residual structures nested in the CSPBlock module, which not only contributes to the problem of gradient disappearance but also improves the performance of the model by deepening the number of convolution layers more stably [20]. The framework of the maritime object detection model in the foggy environment based on SRC-YOLO is shown in Figure 1.

The backbone feature extraction network will output two feature maps of 13 × 13 and 26 × 26 with different scales when the input image size is 416 × 416 × 3 and transfer them to the Neck section. After that, the following three operations are performed on the feature maps in the Neck part in turn. At first, the CBAM is attached to both feature layers that are output from the backbone network separately so that the model can pay more attention to the detailed information in the image. Later, the improved RFB_sim module is employed to process the feature layer in order to improve the model’s receptive field. At the same time, one CBL operation will be performed on the 13 × 13 feature layer, followed by an up-sampling, and then stacked with the 26 × 26 feature layer to enhance the algorithm’s accuracy for small object detection using the fusion of features at different scales. Finally, the class and confidence information of the object will be output directly in the image by the prediction module.

### 2.2. Single Scale Retinex

The dominant image defogging algorithms include Single Scale Retinex (SSR) [21], Automatic Color Equalization (ACE) [22], Multi-Scale Retinex (MSR), Retinex with Color Equalization (MSRCR) [23], and Dark Channel Prior Defogging [24]. In this paper, we conducted comparison experiments on the above five defogging algorithms and finally selected the single scale algorithm with a better dehazing effect to preprocess the data set.

The SSR algorithm is one of the dehazing methods based on image enhancement proposed by Jobson et al. in the Retinex theory. The image presented by the visual system is represented as the product of the incident image and the reflected image in the Retinex theory, which can be shown as Equation (1):

(1) S(x,y)=L(x,y)×R(x,y)

where S(x,y) denotes the original image, L(x,y) represents the incident component, and R(x,y) indicates the reflected component. The main idea of the algorithm is to remove the interference information of the incident image as much as possible and to obtain the reflected image that can show the essential information of the object.

For the convenience of calculation, the logarithmic formula usually substitutes the multiplication formula in Equation (1) in the Retinex theory. The equation is calculated by Formula (2):

(2) logS=log(L×R)=logL+logR

Analysis of a large amount of experimental data shows that the incident component L(x,y) can be approximated using the convolution of a Gaussian function with the original image. As shown in Equation (3):

(3) L(x,y)=S(x,y)∗G(x,y)

(4) $$G\left(x,y\right)=\lambda \exp\frac{-(x^{2}+y^{2})}{c^{2}}$$

where G(x,y) represents the Gaussian function. Additionally, the mathematical expression can be calculated using Equation (4). In the Gaussian function, *c* denotes the Gaussian kernel, which controls the retention of image details and colors, while λ means the normalization constant.

In summary, we can approximately calculate the reflection component of the image through Formula (5):

(5) logR(x,y)=logS(x,y)−log(S(x,y)∗G(x,y))

**Figure 2:**
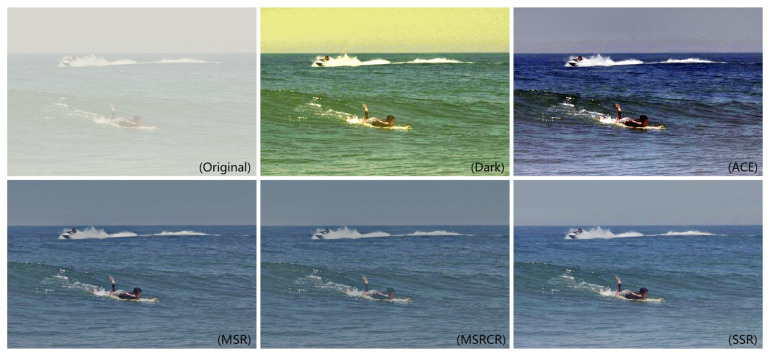
The comparison of dehazing effects with various algorithms.

The performance of distinct defogging algorithms is shown in Figure 2. It is obvious that although the dark channel and ACE algorithms have a dehazing effect on the original image, the overall quality of the image becomes extremely poor. On the contrary, the SSR algorithm has a much better result than the above two algorithms. At the same time, compared with MSR and MSRCR, the image after dehazing by the SSR algorithm is clearer and brighter. In addition, the amount of calculation is less than the former. Therefore, in this paper, we chose the SSR algorithm to perform preprocessing operations on the input images to reduce the interference of the complicated conditions on the model detection. Furthermore, the subsequent experiments also confirm the superiority of the SSR algorithm compared with others.

### 2.3. Improved Receptive Field Block

In the development process of object detection, the acquisition of features with rich global information and high semantic level by increasing the receptive field was widely deployed. There are common ways to enhance the receptive field, such as pooling and dilated convolution. The RFB module, which combines the inception [25] and dilated convolution to simulate the human visual perceptual system, strengthens the feature extraction capability of the neural network by increasing the receptive field [26]. More importantly, RFB has high generalization performance. It has achieved high performance in both SSD [27] and MobileNet [28] in previous experiments. While its main idea is to extract features at different scales of the feature map and perform pooling operations, it can guarantee production of the same scale output for any size of the input image. In this way, it can not only play the role of expanding the receptive field but also extract features in a larger area to extract more feature details, which is more beneficial to the detection of small targets [26].

**Figure 3:**
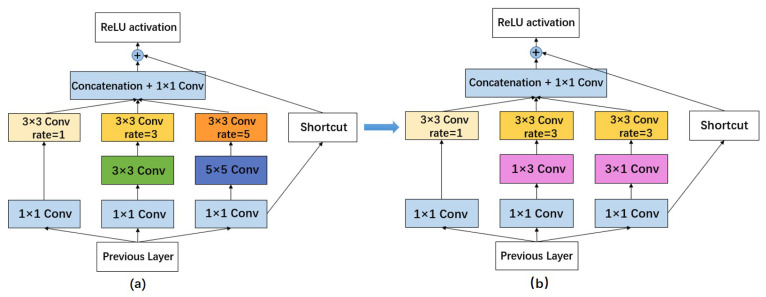
The architecture of RFB and RFB_sim. (**a**) The structure of RFB; (**b**) The architecture of RFB_sim.

The original RFB structure diagram is shown in Figure 3a, combining multiple branches with different kernels and dilated convolution layers, while integrating a residual structure at the perimeter.

The (b) diagram in Figure 3 is the RFB_sim, which is an improved structure based on the RFB. A total of two improvements have been implemented. Firstly, the green 3 × 3 convolution of the middle layer, where rate = 3 is replaced by two convolutions of 1 × 3 and 3 × 1 to reduce the computational effort, which we can see the change from the two pink parts. Secondly, due to the small size of the feature layer, the convolutional branch with rate = 5 is also removed from the model directly to improve the model’s detection speed further. Therefore, only the parts with rate = 1 and rate = 3 are retained in the improved structure.

### 2.4. Convolutional Block Attention Module

Attention mechanism has been validated as an essential approach to effectively prove the ability of feature extraction in convolutional neural networks. In recent years, various forms of attention mechanisms have been proposed one after another, with the central point being to allow the network model better focus on where it needs to pay attention [29,30]. Adding the attention mechanism module to the object detection model can make it focus well on the target information in the image in the complicated and changing marine conditions. Common attention mechanisms are mainly divided into three ways: channel attention, spatial attention, and simultaneous channel and space attention, such as SENet [31], ECA [32], CBAM [33], etc.

**Figure 4:**
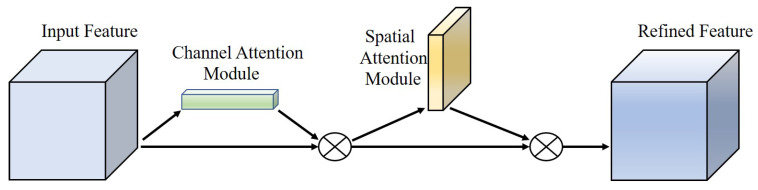
The structure of Convolutional Block Attention Module.

Based on the comparative analysis of the above three attention mechanisms, the CBAM approach, which attaches attention to both independent dimensions of channel and space, is adopted eventually. The structure of CBAM is shown in Figure 4. In particular, the priority of the channel attention and spatial attention module also affects the model’s performance.

## 3. Experimental Results and Analysis

### 3.1. Production of Dataset

Since there is a lack of ocean target image data in foggy conditions, it is necessary to simulate and produce the ocean datasets required for the experiment. We selected 3828 ocean images containing boats and people in the water from TinyPerson, COCO datasets, and UAV ocean aerial videos according to diverse backgrounds, angles, weather, and target sizes in the paper.

**Figure 5:**
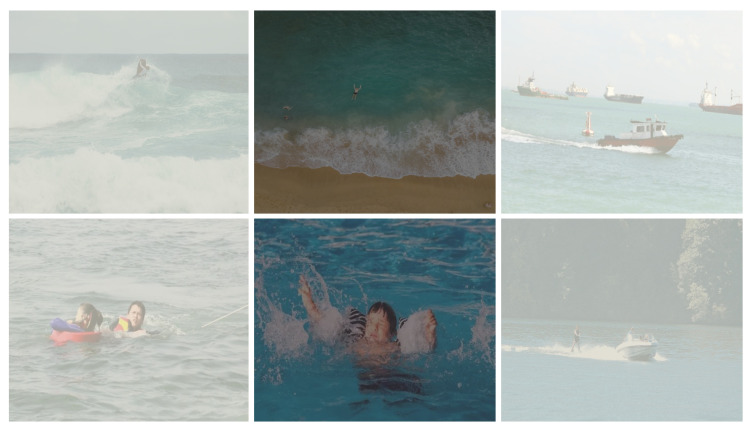
The partial dataset in foggy scenes.

To generate a dataset simulating the foggy climate and improve the robustness of the training model, we employed a hazing algorithm to increase the fogging of the images to different degrees. In addition, the dataset was divided into the training set, validation set, and test set in the ratio of 8:1:1. Finally, the newly released online image labeling tool MakeSense was applied to annotate the objects in the dataset and stored the results in XML format. Figure 5 shows part of the data set in the simulated foggy conditions.

### 3.2. Experimental Environment Configuration and Training Parameter Settings

The main hardware configuration of the training is the Intel Core i7-10700 @ 2.90 GHz processor, and the GPU graphics card is NVIDIA GeForce RTX 2080 SUPER. The application software adopts the deep learning framework PyTorch 1.10, CUDA version 10.2, and Python 3.8. In this experiment, the pictures’ sizes were preprocessed to 416 × 416 × 3 before training.

Since there was no improvement to the backbone feature extraction network of the model, we loaded the pre-training weights of the original YOLOv4-tiny during the training to speed up the model training by the idea of transfer learning. The training process consists of the freezing and unfreezing stages, and the experimental parameter settings are shown in Table 1. In the frozen phase, since the backbone feature extraction network did not change and only fine-tuned the network, thus occupying a relatively small amount of graphics card memory, the batch size at this time was much larger than in the unfreezing stage.

### 3.3. Evaluation Metrics for Model Performance

In the field of object detection, there exists some significant indicators that are usually considered the key metrics to discriminate the performance of a convolutional neural network, such as precision, recall, mean average precision, $F_{1}$ score, FPS, and intersection over union (IOU). Precision indicates the number of positive samples predicted by the model as a percentage of the total samples, as shown in Formula (6). Additionally, recall is the percentage of the total positive samples that are correctly identified, which is calculated by Equation (7). As a matter of fact, precision and recall are a pair of contradictory metrics in general, with higher precision and lower recall. The $F_{1}$ score, the harmonic average of precision and recall, can be utilized for the best combination of precision and recall. The $F_{1}$ score is shown as Equation (8):

(6) $$Precision=\frac{TP}{TP+FP}$$

(7) $$Recall=\frac{TP}{TP+FN}$$

(8) $$F_{1}=\frac{2Precision*Recall}{Precision+Recall}$$

where TP, FP, and FN denote true positive, false positive, and false negative, respectively. In other words, TP represents the number of positive samples with correct classification and FP reflects the number of negative samples incorrectly predicted as positive samples. At the same time, FN indicates the number of positive samples incorrectly predicted as negative samples.

AP is also a primary evaluation criterion that can fully reflect the precision and recall rate, expressing the region value surrounded by the P-R curve with the coordinate axis, as shown in Formula (9). In addition, mAP is the average of the object AP of all classes and is calculated using Equation (10):

(9) $$AP=\sum_{i=1}^{n-1}\left(r_{i+1}-r_{i}\right)p_{i+1}=\int_{0}^{1}p\left(r\right)dr$$

(10) $$mAP=\frac{\sum_{i=1}^{S}AP_{i}}{S}$$

where $r_{1},r_{2}$, …, and $r_{n}$ are the values of recall corresponding to different precisions, respectively. Additionally, *S* indicates the number of object categories to be detected. In this experiment, *S* is 2 for the boat and the person.

The frame rate is a description of the model detection speed. FPS in Equation (11) indicates the number of images that the model can process per second, where time means the amount of time it takes to handle a single image.

(11) $$FPS=\frac{1.0}{time}$$

IOU measures the degree of overlap between the predicted bounding box and the ground truth in object detection. The higher the IOU value, the higher the degree of coincidence between the bounding box and ground-truth box, indicating that the model predicts more accurately. It can be illustrated by Equation (12):

(12) $$IOU=\frac{A⋂B}{A⋃B}$$

where *A* and *B* represent the areas of the bounding box and the ground truth, respectively. The following experiments were all performed with IOU = 0.5 by default.

### 3.4. Experimental Results

**Figure 6:**
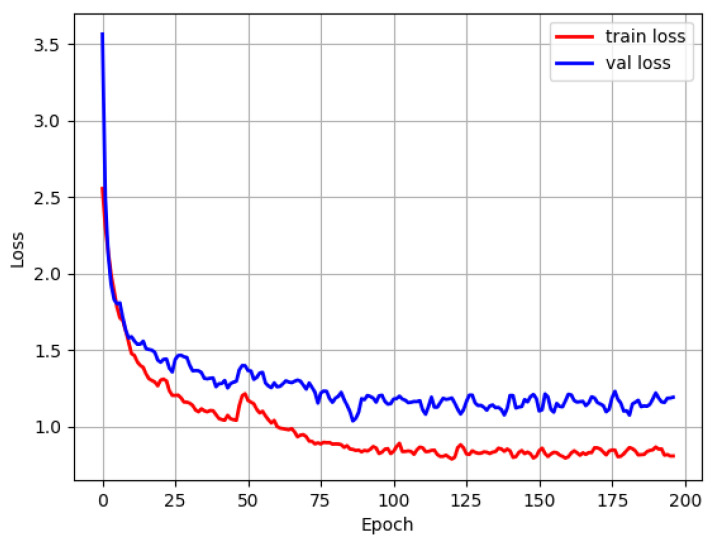
The loss function diagram for model training.

With the improvement of YOLOv4-tiny, the Single Scale Retinex algorithm was first applied to preprocess the input images, followed by the improved RFB_sim module and the convolutional block attention module for the experiments separately. Figure 6 displays the training and validation sets’ loss functions. Since the backbone network employed the pre-training weights, the loss values had a slight jump for a period at the end of the freeze phase, or when the training had completed 50 epochs. After running the training for approximately 100 epochs, the model eventually reached a stable state.

To verify the feasibility of the algorithm, we examined various defogging techniques based on the original YOLOv4-tiny for trials. Table 2 shows that following data preprocessing, the model’s precision, recall, and mAP all greatly improved. In addition, the mAP in this paper indicates the map@.5 by default when IOU adopts a value of 50%. Following the implementation of the SSR method, among them, the mAP increased from 79.56% to 83.81%, or about four percentage points more, as anticipated from the experiment.

The improved RFB_sim module was employed in the second series of trials to increase the model’s receptive field. The experimental outcome of introducing RFB_sim on the basis of data augmentation is displayed in Table 3 below. With the inclusion of only a few parameters, the mean average precision increased still further to 84.82%.

Finally, experiments comparing SENet, ECANet, and CBAM—three distinct self-attention mechanisms—were carried out. The findings demonstrate the superiority of the CBAM with the combination of both channel and spatial attention modules. The addition of the CBAM just marginally lowers the FPS, increasing the mean average precision from 84.82% to 86.15%.

**Figure 7:**
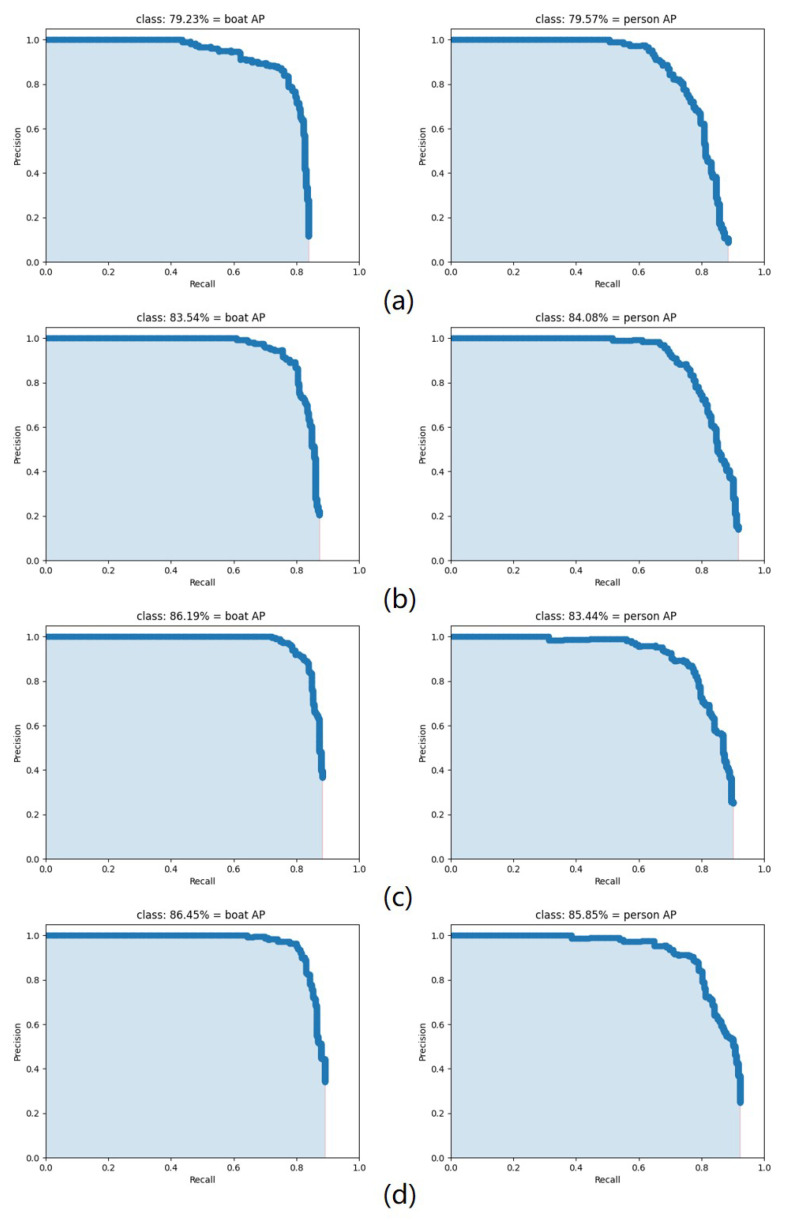
AP curves for the boat and person. (**a**) YOLOv4-tiny; (**b**) YOLOv4-tiny + SSR; (**c**) YOLOv4-tiny + SSR + RFB_sim; (**d**) YOLOv4-tiny + SSR + RFB_sim + CBAM (SRC-YOLO).

Table 4 shows the statistics of the mAP for all the improved strategies in the above ablation experiment. Meanwhile, the AP curves for the boat and human in each group of experimental findings are depicted in Figure 7, respectively. As shown in the table, in each group of improvement experiments, the strategies proposed in this paper have contributed to the model detection accuracy. The introduction of SSR reduces the interference of the complex circumstances on the images, increasing the AP values of the boat and human by 4.31% and 4.51%, respectively. After that, the RFB_sim is adopted to improve the overall mAP value from 83.81 to 84.82%, which is effective in increasing the perceived field of the model. Finally, comparing the results of YOLOv4-tiny + SSR + RFB_sim with SRC-YOLO, it can be seen that the CBAM attention mechanism effectively improves the detection performance of the model, resulting in an increase of mAP to 86.15%.

**Figure 8:**
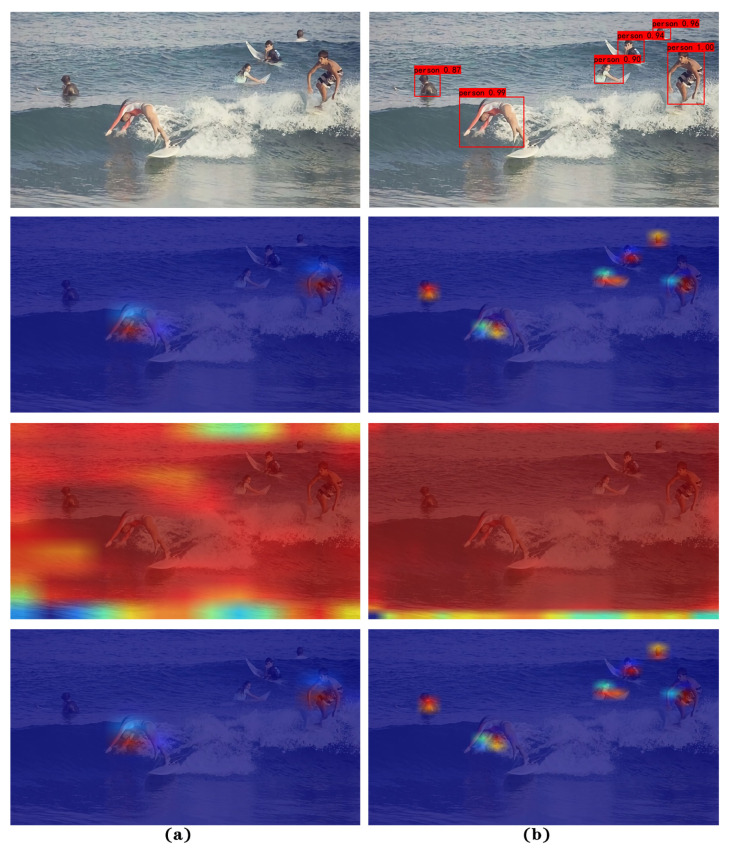
The feature visualization of the SRC-YOLO model. (**a**) The feature visualization of 13 × 13 size feature layer in YOLO head module; (**b**) the feature visualization of 26 × 26 size feature layer in the YOLO head module.

Although the results of the detection can be output directly to the image by an end-to-end approach, the intermediate process of the model remains agnostic, which is equivalent to a black-box model. To explore the performance of feature extraction with the SRC-YOLO network model more intuitively, we visualize it using the CAM [34]. A visualization of the last convolutional layer, which contains the richest spatial and semantic information, is demonstrated in Figure 8. It can be clearly seen from the figure that when the target in the ocean is detected, the model can focus on all the target information in the image by applying the feature layers at different scales. Additionally, SRC-YOLO can accurately identify even small human objects with only their heads exposed to the surface in the distance.

The two visualizations with different sizes in the columns in Figure 8 consist of three diagrams each. Areas, where any target is present, are highlighted in the first visualization result. The second image shows the class score, where the target area containing the specific category we set tends to be highlighted. The third graph is the weighted output of the previous two pictures, representing all possible targets of a certain category in the input image. Since only the person detection is shown in this figure, the results of the first and third figures are consistent. From the visualization results in column (a), the 13 × 13 size feature map is responsible for detecting larger targets in the image, but it is less effective for small targets. From the results in column (b), the 26 × 26 size feature layer detects the small targets in the image well but does not cover the detection of the large targets completely. The final detection results are output under the combination of two separate scales of feature layers.

**Figure 9:**
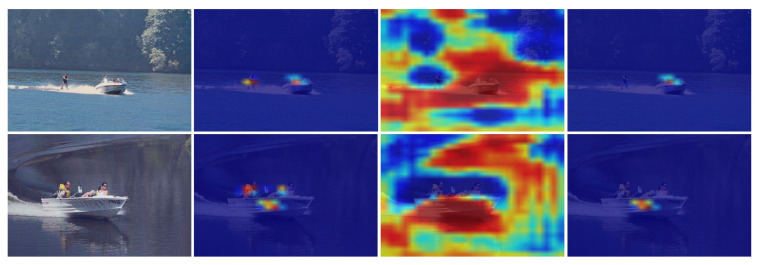
The heatmaps of a ship.

In addition, as we can see in Figure 9, when we set the ships to be detected, the final results can effectively suppress the people in the input images. Heatmap (c) detects all the boats in the figure when (a) and (b) are superimposed. It is the same when detecting people in the image.

**Figure 10:**
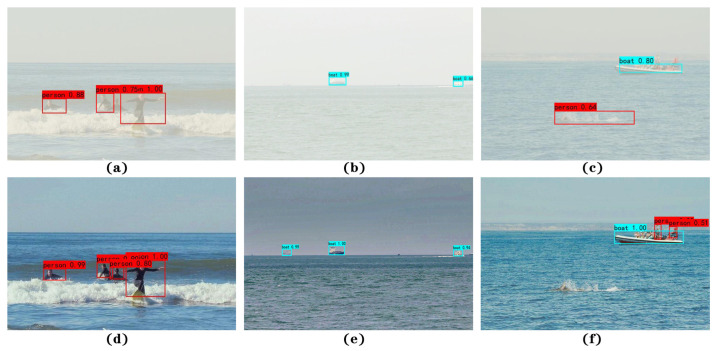
The performance comparison of YOLOv4-tiny and SRC-YOLO. The first row shows the detection results of the original YOLOv4-tiny model, while the second row is for SRC-YOLO. The subplots are the data containing marine boats and people before and after defogging.

Finally, for a better illustration of the detection performance of the SRC-YOLO model proposed in this paper, part of the ocean images under foggy conditions are selected for validation, as shown in Figure 10. From the detection results in the first row of the figure, we can see that the YOLOv4-tiny model has missed detection in all three images due to the impact of the foggy background. For instance, there is only one object detected in Figure 10a, where two people close to each other overlap, and the tiny targets in both Figure 10b,c are not detected either. The worse situation is that the surfaced fish is incorrectly detected as a person in Figure 10c. In contrast, the detection results in the second row indicate that the SRC-YOLO model can precisely identify both the overlapping people and the tiny objects in the distance, including the interference information in the image. In addition, the confidence in the detection is promoted greatly.

## 4. Conclusions

To improve the performance of object detection in complicated conditions, we propose an improved SRC-YOLO model based on YOLOv4-tiny in this paper, which implements the maritime target search and rescue mission in a foggy environment and attains a higher level of accuracy. The improved SRC-YOLO model effectively detects marine targets in foggy scenes by increasing the mean Average Precision (mAP) of detection results from 79.56% to 86.15%.

1. The Single Scale Retinex algorithm is applied before the feature extraction of YOLOv4-tiny, which can effectively reduce the interference of a foggy environment on the detection and plays an essential role in the accurate identification and localization of ships and people on the sea.
2. The introduction of the improved RFB_sim module increases the receptive field with the inclusion of only a few parameters. At the same time, it is capable of capturing more detailed feature information, which is beneficial to the detection of small target objects.
3. Finally, the model’s attention to the object is strengthened by introducing the CBAM combining the information in different dimensions of channel and space, leading to further improvement of the model’s performance.

The SRC-YOLO model proposed in this paper improved the performance of maritime target detection in foggy scenes, but there are still some deficiencies that require refinement. For instance, although the single-scale dehazing algorithm works more effectively, it requires a large amount of calculation and has high requirements for hardware configuration. The rapid image enhancement method based on deep learning will be the priority direction of research in future. In the meanwhile, the impact of other interference factors, such as wind, rain, waves, and sunlight, should also be considered. In addition, compared with the YOLOv4-tiny model, although SRC-YOLO has a better performance in the detection of small objects, there are still some small targets that are missed. Improving the properties of tiny object detection is also one of the directions for future work.

## Figures and Tables

**Table 1:** The parameters at different stages of mode.

Training Stage	Epoch	Batch Size	Learning Rate
Freezing stage	50	32	0.001
Unfreezing stage	150	16	0.0001

**Table 2:** Experimental results of various dehazing algorithms.

Model	Precision/%		Recall/%		*F*_1_ Score		mAP/%
	Boat	Person	Boat	Person	Boat	Person	
YOLOv4-tiny	90.40	86.71	69.57	68.13	0.79	0.76	79.56
YOLOv4-tiny + MSR	89.53	87.50	74.35	73.08	0.81	0.80	82.03
YOLOv4-tiny + ACE	91.62	87.42	76.09	72.53	0.83	0.79	82.66
YOLOv4-tiny + Dark	93.01	89.26	75.22	73.08	0.83	0.80	83.03
YOLOv4-tiny + MSRCR	91.94	87.42	74.35	72.53	0.82	0.79	83.19
YOLOv4-tiny + SSR	91.58	88.24	75.65	74.18	0.83	0.81	83.81

**Table 3:** Experimental results of adopting RFB_sim and various attention mechanisms. The first row indicates the experimental data with the introduction of the RFB_sim module, and the rest indicate the results of different attention mechanisms based on the introduction of RFB_sim.

Model	Precision/%		Recall/%		*F*_1_ Score		mAP/%	Size/MB	FPS/s^−1^
	Boat	Person	Boat	Person	Boat	Person			
RFB-sim	93.85	88.82	79.57	74.18	0.86	0.81	84.82	27.74	106.9
SENet	94.87	87.82	80.43	75.27	0.87	0.81	84.49	27.90	99.1
ECA	95.38	87.26	80.87	75.27	0.88	0.81	85.18	27.74	97.0
CBAM	93.91	89.81	80.43	77.47	0.87	0.83	86.15	28.40	93.5

**Table 4:** The mAP results of ablation experiment.

Model	AP/%		mAP/%
	Boat	Person	
YOLOv4-tiny	79.23	79.57	79.40
YOLOv4-tiny + SSR	83.54	84.08	83.81
YOLOv4-tiny + SSR + RFB_sim	86.19	83.44	84.82
YOLOv4-tiny + SSR + RFB_sim + CBAM(SRC-YOLO)	86.45	85.85	86.15

## Data Availability

Not applicable.
